# Attachment Gain After Applying Xenogeneic Acellular Dermal Matrix in the Management of Isolated Recessions: A Randomized Clinical Trial

**DOI:** 10.7759/cureus.64414

**Published:** 2024-07-12

**Authors:** Mohamad Alabed, Suleiman Dayoub, Mohamad Fawaz

**Affiliations:** 1 Periodontology, Damascus University, Damascus, SYR; 2 Dentistry, International University for Science and Technology (IUST), Damascus, SYR; 3 Public Health, Central Michigan University, Mount Pleasant, USA

**Keywords:** relative attachment level, coronally advanced flap, connective tissue graft, mucoderm, xenogeneic dermal matrix, attachment gain

## Abstract

Background and objectives

Mucogingival plastic surgery is a surgical procedure performed to prevent or correct anatomical, developmental, or traumatic defects. The problem of gingival recession is common in dental practice, causing pain and dentin hypersensitivity for the patient, and remains difficult to treat surgically at the second surgical site used to harvest the connective graft. Many alternatives have been used to replace connective grafts, but none have been as effective. The importance of guided tissue regeneration remains to gain attachment because it means the formation of new periodontal tissue. This study aims to evaluate the attachment gain (AG) obtained after the management of single gingival recessions of Class I and Class II of Miller's classification.

Material and methods

This study was designed as a clinical randomized trial using a split-mouth technique. The study sample included 15 patients (30 symmetrical gingival recessions). The first group included the coronally advanced flap (CAF) with the connective tissue graft (CTG), and the second group included the CAF with the Xenogeneic Acellular Dermal Matrix (XDM) (Mucoderm®, Botiss Biomaterials, Zossen, Germany). AG was measured at baseline and after six months.

Results

The results showed that the mean relative attachment level at baseline was 8.333±0.899 in the CTG+CAF group and 8.466±0.833 in the XDM+CAF group. After six months of follow-up, the levels remained 8.333±0.899 in the CTG+CAF group and 8.466±0.833 in the XDM+CAF group, with a significant difference between the study groups after six months.

Conclusion

The current study concluded that both grafts applied with the coronally advanced flap led to a gain in attachment, with a greater gain in the CTG group.

## Introduction

Mucogingival plastic surgery is a surgical procedure performed to prevent or correct anatomical, developmental, or traumatic defects or diseases caused by gingival, alveolar mucosa, or bony defects. Gingival recession is one such defect that affects both the functional and aesthetic aspects of the patient [1].

The primary goal of mucogingival plastic surgery is to obtain complete root coverage, treat gingival recession while maintaining minimal probing depth after treatment, and form a new attachment with the tooth, which is essential in determining the treatment results. It also aims to achieve sufficient width and thickness to prevent mucogingival defects [2].

Various techniques have been used to manage localized and multiple gingival recessions; some rely on the use of advanced flaps in all their forms, while others involve a combination of advanced flaps with autologous and/or synthetic grafts [3]. To date, the treatment option combining a coronally advanced flap (CAF) with a connective tissue graft (CTG) harvested from the palate is considered the gold standard in managing gingival recessions [4].

However, autogenous gingival grafts suffer from several limitations due to the variable thickness of soft tissue in the palate and pain following surgery caused by secondary surgical trauma [5].

These limitations have led researchers to evaluate the effectiveness of synthetic grafts and substitutes such as enamel matrix derivative (EMD), acellular dermal allograft (Alloderm), platelet-rich plasma (PRF), and collagen membranes (Mucograft, Mucoderm, Fibro-Gide) as alternatives to CTGs in managing gingival recessions and treating mucosal defects [6-7]. Collagen membranes are used to build periodontal tissue through a process known as guided tissue regeneration and have shown good clinical results in treating bone defects; they are now also used in treating gingival recessions [8].

This study utilized a Xenogeneic Acellular Dermal Matrix (XDM) (Mucoderm®, Botiss Biomaterials, Zossen, Germany), a xenogeneic acellular collagen matrix with a three-dimensional structure composed of a single dense layer of type I and III collagen fibers [9,10].

This study aims to evaluate the attachment gain (AG) obtained after managing a single gingival recession of Class I and Class II of Miller's classification.

## Materials and methods

Study design

**Figure 1:**
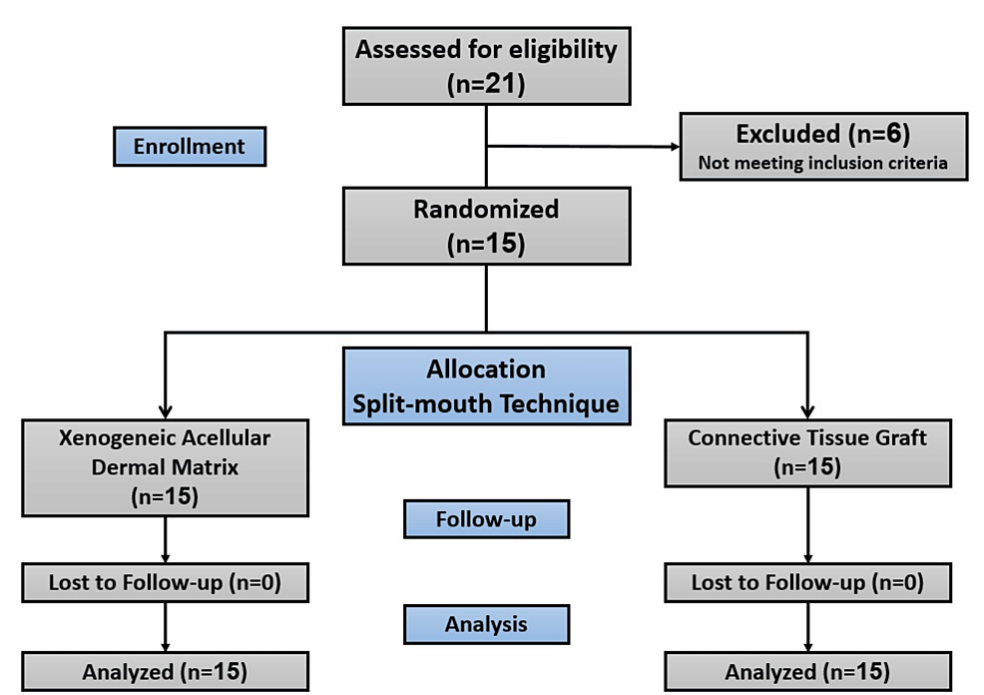
CONSORT flow chart of patients.

This study was designed as a clinical randomized trial using a split-mouth technique. The study sample included 15 patients with 30 symmetrical gingival recessions. The allocation ratio of the study was 1:1 (Figure 1), where the first group included the CAF procedure with the application of the CTG, which served as the control group, and the second group included the CAF with the application of the Xenogeneic Acellular Dermal Matrix (XDM) (Mucoderm®, Botiss Biomaterials, Zossen, Germany).

This study was registered in the BMC Clinical Trials Registration database with ID (ISRCTN15571664), following the Helsinki Declaration and the CONSORT statement. All patients evaluated in the study were informed of the benefits and risks of participating in the research and signed written informed consent before inclusion.

The study sample was randomized using Excel software. Clinical measurements were made by a single-blinded researcher (Fawaz M), and surgical procedures were performed by another researcher (Alabed M).

Eligibility criteria

The study sample included patients with isolated, symmetrical gingival recession of Class I or II according to Miller's classification in the anterior teeth, who were systemically healthy and had good oral health (plaque score less than 20%), did not have active periodontal disease, were non-smokers, and consented to join the study after signing informed consent.

Patients with diseases affecting healing, pregnant women, and lactating women were excluded from this study. Those with multiple or adjacent recessions to edentulous areas were also excluded from the study sample.

Clinical measurements

**Figure 2:**
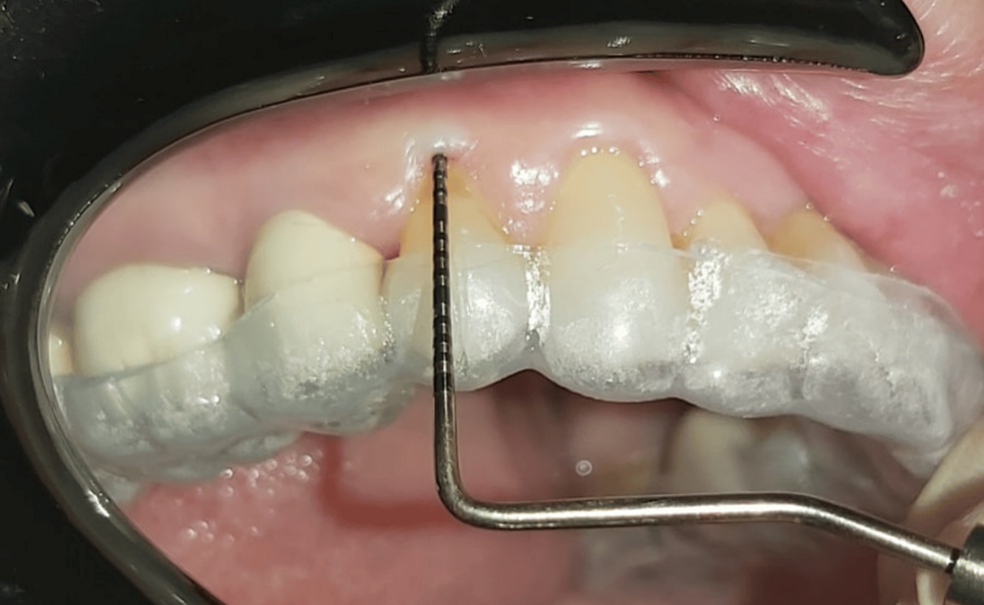
Relative attachment level measurement using acrylic stent.

Relative attachment level (RAL) was measured from the base of the gingival sulcus to the margin of a custom-designed stent (acrylic stent) (Figure 2) at baseline and after six months of the surgical procedure.

AG is the difference between the RAL at baseline and after six months. It is calculated as follows: AG = RAL at Baseline - RAL at 6 months.

All previous clinical measurements were performed using a periodontal probe (UNC15, JK Surgical, Pakistan).

Pre-surgical therapy

All enrolled patients underwent initial treatment, which included necessary scaling and root planning. A periodontal re-evaluation was conducted two weeks after the first phase of treatment.

Surgical procedures

**Figure 3:**
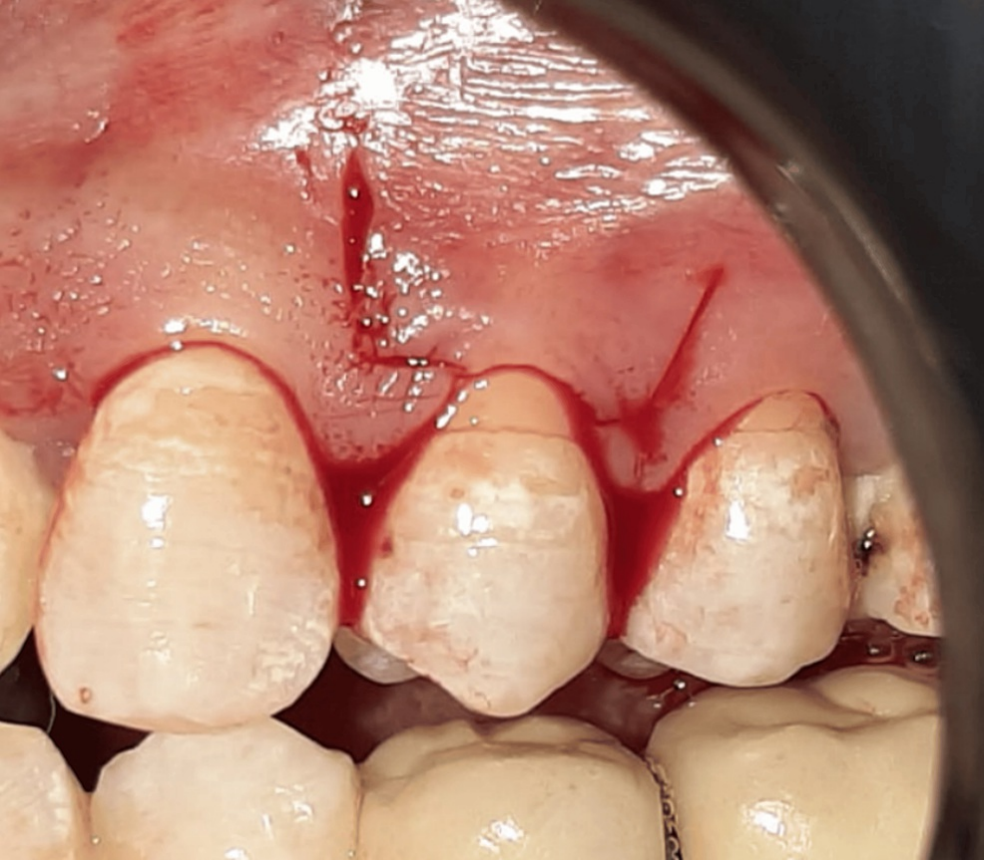
The design of coronally advanced flap.

The surgical procedure in both study groups included the coronally advanced flap, performed by making a sulcular incision and two horizontal incisions in the gingival papilla, distanced from the apex of the papilla by the gingival recession + 1 mm. Two vertical incisions were then made to release the flap using a 15C blade according to the protocol described in (Figure 3) [5]. The remaining gingival papilla was de-epithelialized.

After preparing the recipient site, one of the procedures (CTG or XDM) was applied as follows:

CTG

Harvested from the palate as a free, full-thickness gingival graft from the first molar area. The epithelium was removed from the free gingival graft outside of the oral cavity using a No. 15 blade, the fatty layer was removed from the interior of the graft, and it was thinned until it was 1-1.25 mm in thickness.

The connective graft was fixed at the recipient site by suturing it to the gingival papillae with 6-0 absorbable interrupted suture (PLGA thread, Vertmed, Germany) at the level of the cemento-enamel junction.

XDM (Mucoderm)

**Figure 4:**
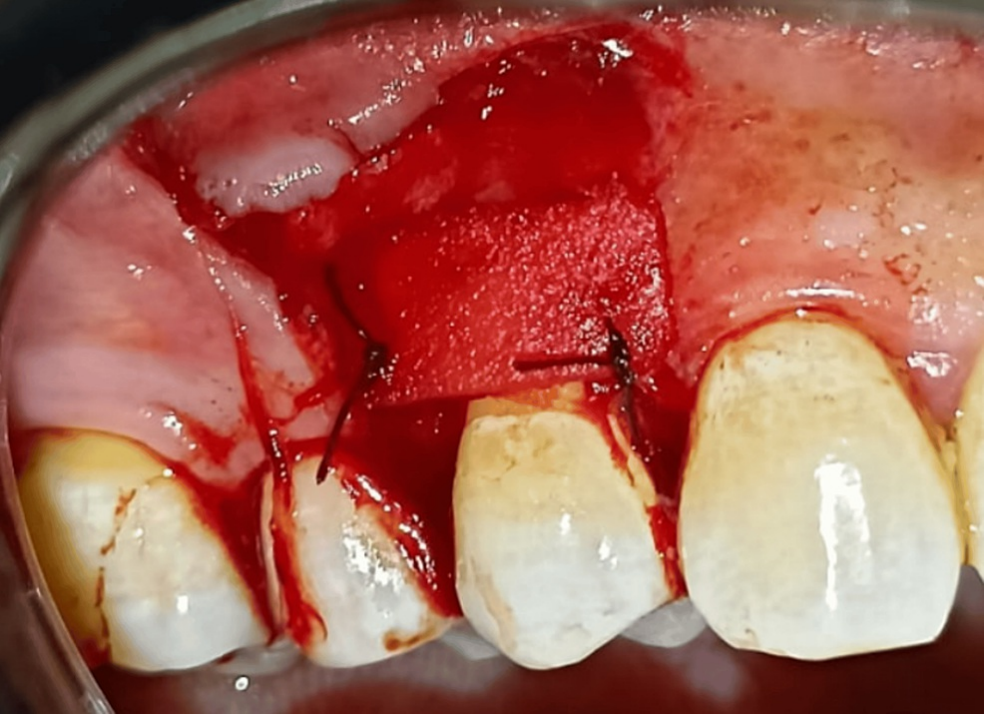
The graft sutured to the recipient site (same method in both groups).

Hydrated by soaking in saline for 15 minutes, then adapted to the recipient site and fixed by suturing it to the gingival papillae with 6-0 absorbable interrupted suture (PLGA thread, Vertmed, Germany) at the level of the cemento-enamel junction as shown in Figure 4.

**Figure 5:**
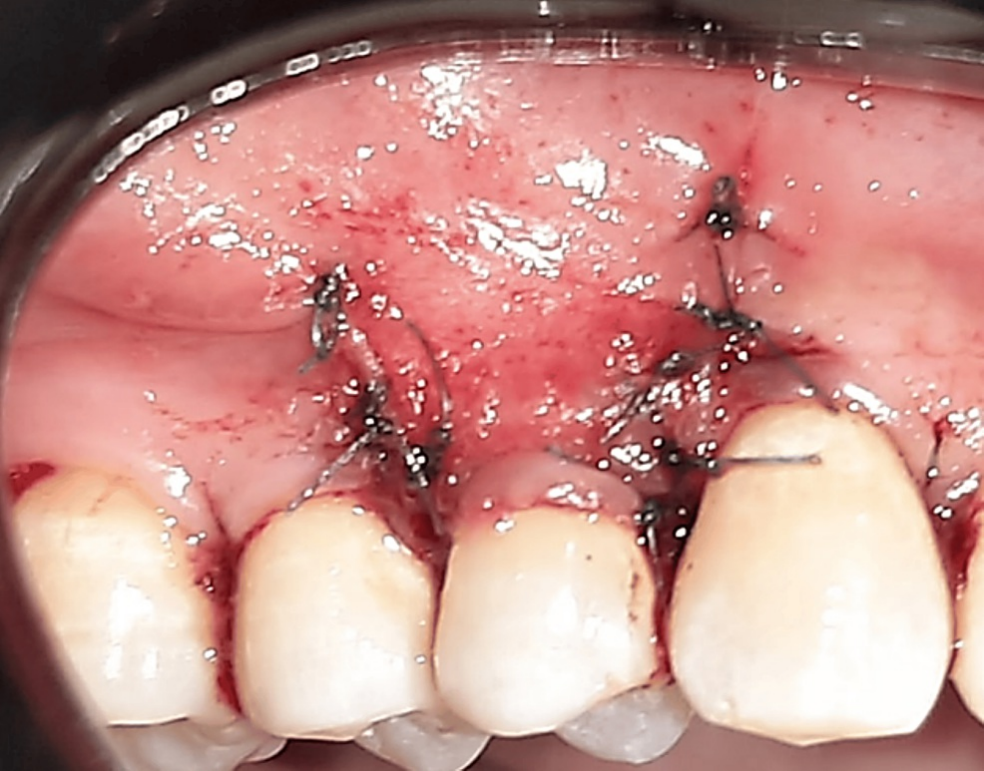
The suture of the coronally advanced flap.

The gingival flap in both groups was fixed by suturing it 1 mm above the cemento-enamel junction with a sling suture, and the vertical incisions were sutured with a single interrupted suture using 6-0 thread (Nylon thread, Vertmed, Germany) as shown in Figure 5.

No medication was prescribed post-surgery. Patients were instructed to adhere to a soft diet during the first week of surgery and to use 0.12% chlorhexidine rinse twice a day for a week.

Sutures were removed 14 days after surgery, and clinical measurements were evaluated six months later.

The surgical interventions were performed a month apart between the two sites in the same patient.

Statistical analysis

Data were collected by a single examiner (Fawaz M), entered into Excel, and statistically analyzed using SPSS version 26 (IBM, SPSS Inc, USA).

## Results

The study sample consisted of 15 patients (30 surgical sites) with an average age of 26.2±4.2 years. The sample was divided into six female patients and nine male patients as detailed in Table 1.

**Table 1:** Demographic analysis of study sample. CTG: Connective tissue graft; CAF: Coronally advanced flap; XDM: Xenogeneic Acellular Dermal Matrix.

Group	Number of extraction sites	Male	Female	Age mean
CTG+CAF	15	9 (60%)	6 (40%)	26.2±4.2
XDM+CAF	15			

The results showed that the mean RAL at baseline was 8.333±0.899 in the CTG+CAF group and 8.466±0.833 in the XDM+CAF group. After six months of follow-up, it was 6.400±0.985 in the CTG+CAF group and 6.866±0.915 in the XDM+CAF group (Table 2).

**Table 2:** Mean of relative attachment level. CTG: Connective tissue graft; CAF: Coronally advanced flap; XDM: Xenogeneic Acellular Dermal Matrix; RAL: Relative attachment level.

Measurements	Number	CTG+CAF	XDM+CAF
RAL Baseline	15	8.333±0.899	8.466±0.833
RAL Six Months	15	6.400±0.985	6.866±0.915

The Wilcoxon test, a nonparametric comparison test, was performed to assess differences in the relative attachment levels between the two study groups. At baseline, the p-value was 0.175, indicating no statistically significant difference. After six months of follow-up, the p-value was 0.025, showing a statistically significant difference between the two groups (Table 3).

**Table 3:** Results of the Wilcoxon test for relative attachment levels. *P-value < 0.005 indicates a significant difference.

Measurements	Number	Wilcoxon Test P-value
RAL Baseline	15	0.175
RAL Six Months	15	0.025*

The mean attachment gain was 1.933±0.785 in the CTG+CAF group and 1.600±0.655 in the XDM+CAF group.

After conducting the Wilcoxon comparison test, the p-value was 0.008, indicating a statistically significant difference in favor of the CTG+CAF group (Table 4).

**Table 4:** Mean of attachment gain and Wilcoxon test results. *P-value < 0.005 indicates a significant difference. CTG: Connective tissue graft; CAF: Coronally advanced flap; XDM: Xenogeneic Acellular Dermal Matrix.

Measurements	Number	CTG+CAF	XDM+CAF	Wilcoxon Test P-value
Attachment Gain	15	1.933±0.785	1.600±0.655	0.008*

The study results revealed a gain in attachment for both groups, with a noticeable advantage for the CTG group after six months. The improvement in both groups may be attributed to a reduction in gingival recession while maintaining consistent probing depth.

## Discussion

Many synthetic grafts have been applied in combination with the CAF, among which collagen grafts are particularly significant due to their ability to enhance the differentiation and proliferation of specialized cells in the area, in addition to promoting blood flow and wound healing [11].

Due to the biocompatibility of collagen grafts in periodontal surgery, they have multiple uses. A three-dimensional collagen matrix (Mucoderm) characterized by a thickness of 1.2-1.7 mm is currently available. Its matrix includes a single layer of high-density collagen that ensures the stability of the blood supply and enhances the regeneration of periodontal tissue. This collagen matrix is used in managing gingival recessions and increasing gingival mucosa dimensions because it is easy to use and apply [12].

The results of this study showed an AG in both groups, with a significant difference in favor of the connective tissue graft group after six months. This gain in both groups can be explained as a result of a decrease in gingival recession while the probing depth remains constant since the healing after root coverage is represented by the formation of a new attachment on the root side and the formation of a long epithelial attachment on the coronal side. Histological studies are needed to confirm these results [13].

Until now, there has been one clinical study that evaluated the AG in single recessions using a xenogeneic collagen matrix [14], and some studies have evaluated it in multiple recessions [9,15,16].

The study by Santamaria MP et al., (2022) [14], compared two types of CTG alternatives. The first group included the CAF alone, the second group included the CAF in conjunction with the collagen matrix, and the third group included the CAF with the xenogeneic acellular dermal matrix in treating single recessions. The results of this study showed that there were no significant differences between the three study groups in terms of root coverage and clinical AG.

A study by Meza-Mauricio J et al., (2021) [9], compared Mucoderm and CTG with the CAF in covering multiple recessions of Class I and II Miller with the split-mouth technique in 42 patients, after a follow-up period of 12 months. The results of the study determined a similar AG between the two groups.

A study by Maluta R et al., (2021) [15], compared the use of Mucoderm and CTG with the CAF in covering multiple gingival recessions of Class I and II Miller in 15 patients. After a six-month follow-up period, there were no differences between the two study groups in terms of AG.

A study by Rakasevic DL et al., (2020) [16], compared Mucoderm and CTG with the modified CAF in covering multiple contiguous gingival recessions (Miller Class I) using the split-mouth technique in 20 patients. After a follow-up period of 12 months, both groups showed improvement in the studied clinical parameters, and the results indicated a similar AG between the two groups.

This study has limitations represented by the inability to conduct a histological study on humans to determine the histological differences between the two study groups, which may be useful in improving the alternatives used for CTGs.

## Conclusions

The problem of gingival recession is common in dental practice, causing pain and dentin hypersensitivity for patients. It remains difficult to treat surgically, especially at the second surgical site used to harvest the connective graft. Many alternatives have been tried to replace connective grafts, but none have been as effective. The importance of guided tissue regeneration lies in its ability to facilitate AG, which signifies the formation of new periodontal tissue. The current study concluded that both grafts, when applied with the CAF, led to an AG, with the gain being greater in the CTG group.
